# Acute Total Hip Arthroplasty with or Without Internal Fixation for Acetabular Fractures in the Elderly: A Case Series

**DOI:** 10.3390/medicina62020350

**Published:** 2026-02-10

**Authors:** Vasileios Athanasiou, Vasileios Giannatos

**Affiliations:** Orthopedics and Traumatology Department, University Hospital of Patras, 26504 Patras, Greece; vass.athanasiou@gmail.com

**Keywords:** acetabulum, cotyloidoplasty, acute THA

## Abstract

*Background and Objectives*: Acetabular fractures in elderly patients are increasing in incidence and are frequently associated with osteoporotic bone, fracture comminution, marginal impaction, and pre-existing joint degeneration. Open reduction and internal fixation (ORIF) alone in this population is associated with high rates of fixation failure, post-traumatic osteoarthritis, and secondary conversion to total hip arthroplasty (THA). Acute THA, with or without concomitant internal fixation, has emerged as an alternative strategy aimed at enabling early mobilization and reducing reoperation rates. *Materials and Methods*: We retrospectively reviewed a series of elderly patients who sustained an acetabular fracture and were treated with acute THA, either as a standalone procedure or combined with internal fixation. Demographic data, fracture patterns, surgical technique, implant choice, complications, and short-term clinical and radiographic outcomes were analyzed. *Results*: Acute THA allowed immediate or early weight bearing in all patients. Implant stability was achieved using a highly porous, multi-hole acetabular component with supplemental screw fixation and selective use of internal fixation to restore columnar stability when required. Complications were comparable to those reported in the contemporary literature for acute THA in acetabular fractures. *Conclusions*: In carefully selected elderly patients with acetabular fractures at high risk of failure after ORIF, acute THA with or without internal fixation represents a viable definitive treatment strategy, enabling early mobilization and avoiding the morbidity associated with delayed salvage arthroplasty.

## 1. Introduction

The epidemiology of acetabular fractures has shifted markedly over the past two decades. While historically considered high-energy injuries affecting younger patients, acetabular fractures are increasingly encountered in elderly individuals following low-energy mechanisms, such as falls from standing height, as a consequence of the rising life expectancy [1,2]. Large national registries have demonstrated that more than 60% of acetabular fractures occur in patients older than 70 years old, with reported one-year mortality rates approaching those of proximal femoral fractures, highlighting the systemic impact of these injuries [3,4].

Acetabular fractures in the elderly are frequently characterized by anterior column involvement, quadrilateral plate medialization, superomedial dome impaction (“gull sign’’), and osteoporotic bone, all of which compromise fixation stability and biological healing potential [1,5]. Historically, initial management involved nonoperative treatment with prolonged bed rest; however, this approach is currently reserved for minimally displaced acetabular fractures or patients with significant comorbidities [6]. Open reduction and internal fixation (ORIF) remain the gold standard for displaced acetabular fractures in younger patients. However, outcomes following ORIF in the elderly are less predictable. Reported rates of post-traumatic osteoarthritis following ORIF range from 12% to 57%, with conversion to THA often occurring within the first two years after injury [7,8,9,10]. Radiographic features, including femoral head impaction, marginal dome impaction, posterior wall comminution, and the presence of the “gull sign”, have been shown to predict fixation failure and early conversion to arthroplasty [11,12,13].

Delayed THA following failed fixation is technically demanding and associated with increased operative time, blood loss, and higher rates of infection, dislocation, and revision when compared with acute THA [14,15,16,17]. These observations have prompted a paradigm shift toward acute THA, with or without concomitant internal fixation, in selected elderly patients with acetabular fractures unlikely to achieve durable outcomes with ORIF alone [18,19,20,21]. The new paradigm, called “fix and replace”, prioritizes immediate joint stability and early full weight bearing, trying to avoid fixation failure and secondary conversion surgery; thus, the goal shifts from anatomic reduction to restoration of the relationship between the anterior inferior iliac spine (AIIS) and the ischium [17,19,22,23].

The purpose of this study is to present a case series of elderly patients treated with acute THA with or without concomitant ORIF for acetabular fractures and to contextualize the findings within the contemporary literature.

## 2. Materials and Methods

### 2.1. Study Design and Patient Selection

This retrospective case series (February 2021 to October 2023) included consecutive elderly patients presenting with an acute acetabular fracture who underwent primary THA during the acute post-injury period. Inclusion criteria comprised advanced age (≥60 years), fracture patterns associated with poor prognosis following ORIF, osteoporotic bone, or pre-existing hip osteoarthritis. Patients initially treated with isolated ORIF, who subsequently experienced early failure or required delayed THA, were excluded. Ethical review and approval were waived for this study. According to Greek legislation (Law 3418/2005, Code of Medical Ethics), retrospective case series that utilize anonymized data from medical records and do not involve any new intervention or deviation from standard clinical practice do not constitute “interventional clinical research” requiring formal approval from the National Ethics Committee. Patient data confidentiality was ensured in strict compliance with the General Data Protection Regulation (GDPR) and Greek Law 4624/2019.

### 2.2. Fracture Classification and Preoperative Assessment

Fractures were classified according to the Judet–Letournel system based on plain radiographs and computed tomography with three-dimensional reconstructions. Particular attention was paid to the integrity of the anterior and posterior columns, involvement of the quadrilateral plate, dome impaction, and femoral head injury, as these factors guided the decision to perform THA alone or in combination with internal fixation. The integrity of the AIIS-ischium axis was assessed as the axis represents the principal biomechanical buttress for acetabular component stability. In cases where this axis was disrupted (anterior column, posterior hemitransverse, T-type, transverse, both columns), limited non-anatomical internal fixation was performed.

### 2.3. Surgical Technique

**Figure 1:**
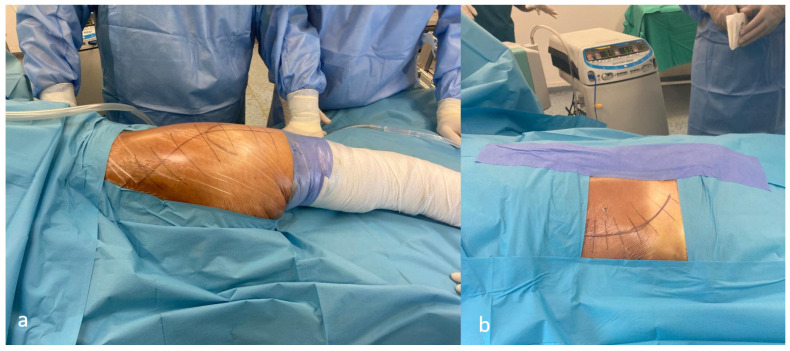
Surgical approaches: (**a**) Kocher–Langenbeck, (**b**) ilioinguinal.

**Figure 2:**
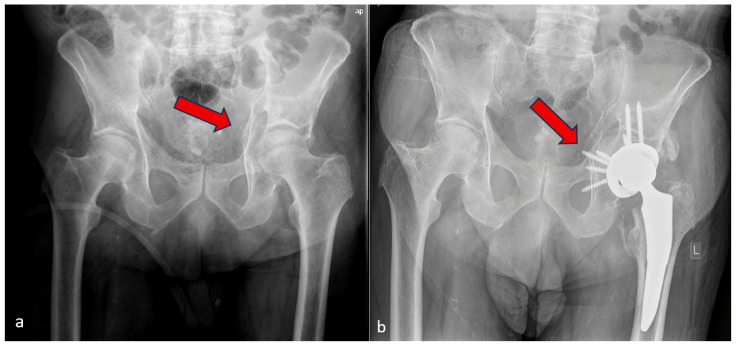
Acetabular fracture with intact anterior and posterior columns: (**a**) pre-operative radiograph showing quadrilateral plate fracture (red arrow), (**b**) one-year post-operative radiograph showing isolated THA augmented with femoral head autograft (cotyloidoplasty—red arrow).

**Figure 3:**
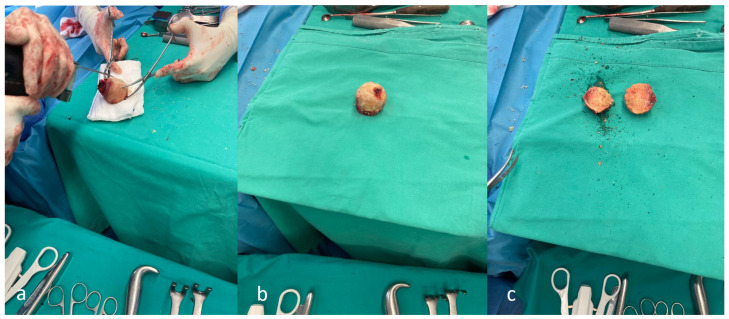
Femoral head autograft preparation (**a**) decortication using an oscillating saw, (**b**) decorticated femoral head, and (**c**) contoured using an oscillating saw and osteotomes to fit the acetabular defect.

**Figure 4:**
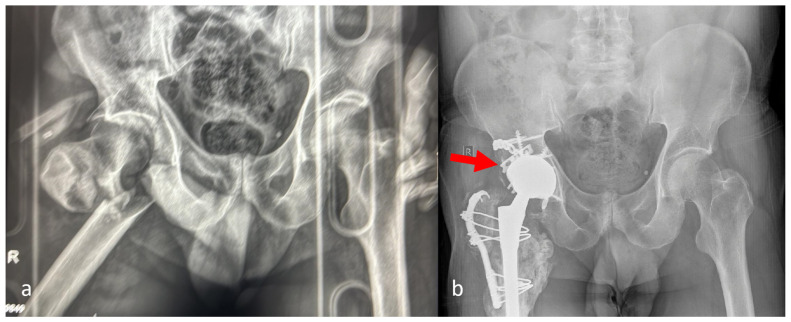
Acetabular fracture (**a**) pre-operatively and (**b**) post-operatively. A reconstruction plate along with two hook spring plates (contoured as per AO principles) have been used posteriorly (red arrow) to stabilize the femoral head autograft.

**Figure 5:**
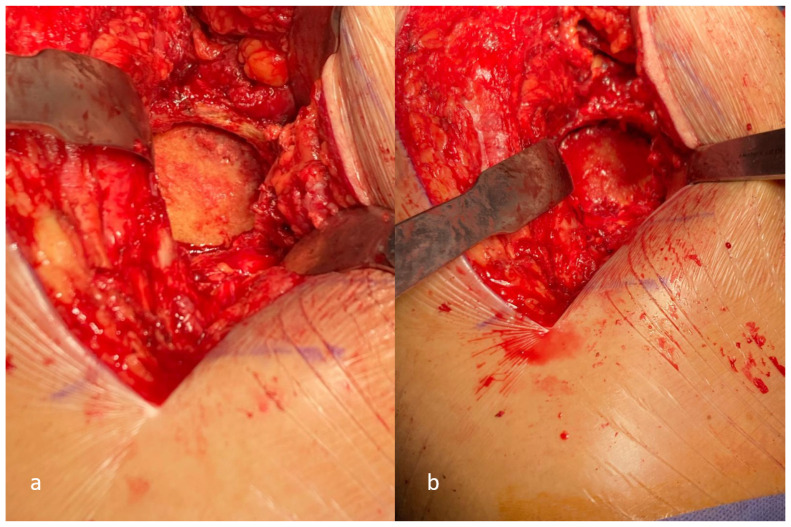
Femoral head autograft decorticated and contoured (**a**) before and (**b**) after reaming.

**Figure 6:**
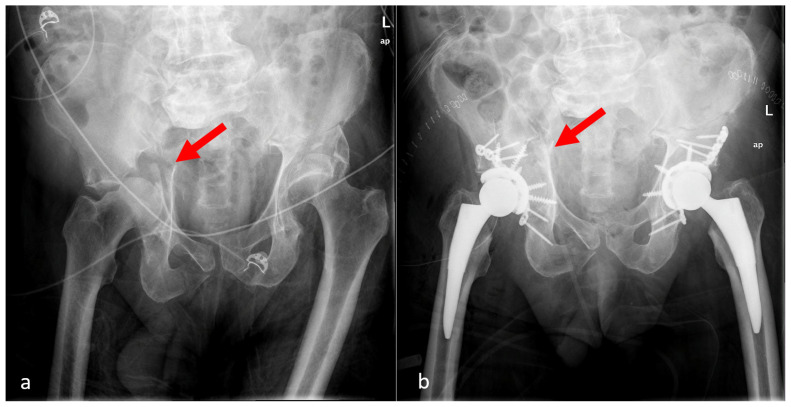
Bilateral fix and replace technique (**a**) pre-operatively and (**b**) post-operatively. The red arrow indicated the non-anatomic reduction in the posterior and anterior columns. A plate and multiple screws in the acetabular shell were utilized to stabilize the femoral head autograft.

**Figure 7:**
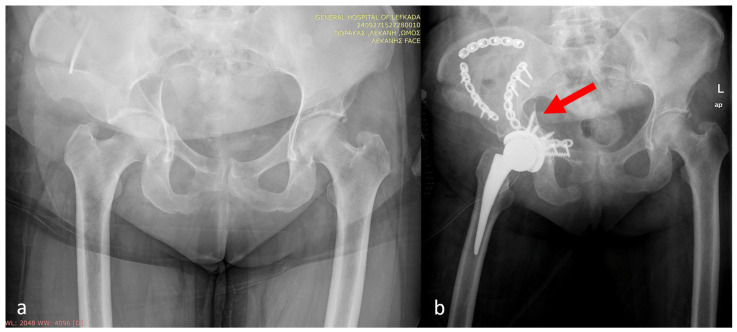
Acetabular and pelvic fracture (**a**) pre-operatively and (**b**) post-operatively. Multiple screws have been used for the shell, femoral autograft fixation, and fracture fixation in a non-anatomic fashion (red arrow).

All procedures were performed by an experienced trauma and arthroplasty surgeon (VA). A single-stage procedure using the Kocher–Langenbeck approach was performed in all cases, except one case where it was combined with an ilioinguinal approach (Figure 1). Following femoral neck osteotomy, the femoral head was temporarily preserved for use as an autograft if required. Although anatomical reduction was not always essential, any major deformity was corrected to restore the overall shape of the acetabulum for cup implantation. The stability of the construct as assessed pre-operatively and intra-operatively was vital, however, in order to achieve implant fixation and ensure longevity. In the case of intact posterior and anterior columns, we opted for isolated THA according to ring stability intra-operatively, dome and medial wall impaction, and the patient’s needs. In case of intact anterior and posterior columns with loss of quadrilateral plate, isolated THA was augmented with cotyloidoplasty, reaming the femoral head in the acetabular floor, and stabilizing it with multiple screws through the multi-hole cup (Figure 2). When ORIF was necessary, standard acetabular reduction techniques using pelvic reduction clamps over cortical screws were employed to reduce displaced fragments and maintain reduction while the plate was applied. In cases requiring reconstruction of the medial or posterior wall, an autograft from the femoral head was utilized. Segmental acetabular defects were reconstructed using a structural autologous femoral head graft. The non-morselized graft was decorticated, contoured, and impacted into the acetabular defect to restore acetabular integrity and provide immediate mechanical stability (Figure 3). Fixation of the non-morselized femoral head autograft was achieved primarily either through a press-fit fashion utilizing a wedge shape wedged into the defect or via screws and plates (Figure 4). Reaming was performed sequentially after fixation of the bone graft to achieve the final acetabular shape (cotyloidoplasty) (Figure 5). A multi-hole cup was used in all cases, including the PINNACLE cup with GRIPTION coating and TRI-LOCK Bone Preservation Stem (DePuy Synthes, Warsaw, IN, USA), as well as tantalum cups with Taperloc stem and Wagner cone stem (Zimmer Biomet, Warsaw, IN, USA) in one case. Cups were implanted either via press-fit (in situ) or secured with 3–5 screws in 2–3 available fixation corridors, augmenting autograft fixation and providing implant stability at the same time (Figure 6 and Figure 7). Intraoperative fluoroscopy (C-arm) was used in all procedures to confirm component positioning and femoral leg length and offset. Dual-mobility cups or large-diameter heads were available and considered for potential use in patients at elevated risk of instability, but they were ultimately not required.

### 2.4. Postoperative Management and Outcomes

Early mobilization with immediate or early weight bearing was encouraged whenever feasible. Clinical outcomes, complications, and radiographic assessment were systematically evaluated during follow-up.

## 3. Results

### 3.1. Patient Demographics and Injury Characteristics

A total of 17 acute THAs were performed in 16 patients (one patient with bilateral acetabular fractures) at a Level I trauma center between 2020 and 2023. The cohort had a mean age of 67.3 ± 6.7 years, reflecting the typical demographic profile of elderly patients sustaining low-energy acetabular fractures. The mean follow-up duration was 2.6 years (1.2–4 years). Most injuries resulted from low-energy mechanisms, primarily falls from standing height, although a minority of cases were associated with high-energy mechanisms, such as falls from greater heights or motor vehicle accidents, consistent with previously reported epidemiological patterns in this population.

Fracture patterns were heterogeneous. The most common configurations included anterior column fractures with posterior hemitransverse extension, both-column fractures, and transverse fractures with associated marginal dome impaction. Quadrilateral plate involvement and medialization were frequently observed on preoperative computed tomography. Six patients demonstrated radiographic features associated with a high risk of failure following ORIF, including femoral head impaction and acetabular dome comminution. Pre-existing degenerative changes in the hip joint were present in a subset of patients and contributed to the decision to proceed with acute arthroplasty rather than isolated fixation.

### 3.2. Surgical Management

All patients underwent acute total hip arthroplasty within the early post-injury period. In 10 patients, THA was performed as a standalone procedure, whereas 6 patients required concomitant internal fixation to restore acetabular column stability prior to component implantation. A highly porous, multi-hole acetabular component was used in all cases, with supplemental screw fixation to enhance primary stability. Screws were directed toward the ilium and ischium in accordance with contemporary biomechanical principles. Femoral head autograft was utilized in four cases in a ‘cotyloidoplasty’ fashion to address contained acetabular defects when present. Internal fixation, when employed, was limited to the minimum necessary to restore structural stability and facilitate secure cup implantation rather than to achieve anatomic fracture reduction. This strategy was consistent with the concept of prioritizing implant stability over fracture anatomy in elderly patients with compromised bone quality.

### 3.3. Postoperative Mobilization

Early mobilization was achieved in all patients. Immediate or early weight bearing as tolerated was permitted following surgery, facilitated by the inherent stability of the arthroplasty construct. No patients required prolonged protected weight bearing, and no secondary immobilization was necessary. At the final follow-up, clinical outcomes were very good, with a mean modified Harris Hip Score (mHHS) of 82.3 ± 5.1.

### 3.4. Complications

Perioperative and early postoperative complications occurred at rates comparable to those reported in contemporary series of acute THA for acetabular fractures. Intra-operatively, one case (5.8%) of external iliac artery injury occurred, diagnosed immediately post-op and treated successfully with an endovascular stent by two experienced interventional radiologists. Two cases (11.7%) of peroneal nerve palsy were recorded post-operatively, with one case recording improvement at the last follow-up. One 80-year-old patient (5.8%) reported a dislocation after a fall from a standing height during the early post-operative period, managed with closed reduction and no recurrent episodes at final follow-up. No cases of early deep infection or catastrophic implant failure were observed during the short-term follow-up period. Radiographic evaluation revealed no early acetabular component migration or loss of fixation as assessed macroscopically by two orthopedic surgeons at the last follow-up, compared with the immediate post-operative evaluation. Clinical symptoms, such as pain at rest or during activities, were also recorded to assess implant migration, with no evidence of migration either. Regarding medical comorbidities, two patients developed pulmonary embolism in the acute post-operative setting, diagnosed by CTPA immediately after symptom onset and treated until full recovery. No deaths occurred as a consequence of the fracture during the follow-up period. Collectively, all implants remained radiographically stable at the last follow-up with no revision surgery required during the follow-up period.

## 4. Discussion

The management of acetabular fractures in the elderly remains challenging. ORIF alone is frequently associated with early failure, prolonged protected weight bearing, and high rates of conversion to THA [7,8,9,10]. National database studies have demonstrated that although acute THA may be associated with higher short-term readmission rates, it confers lower long-term revision risk compared with ORIF alone and earlier post-operative weight-bearing, which is critical for a good post-operative outcome [24,25].

A central principle in the acute arthroplasty management of acetabular fractures is that anatomic fracture reduction is not a prerequisite for successful outcomes, provided that sufficient structural stability is achieved to support acetabular component fixation. This concept represents a departure from the traditional ORIF goals, where anatomic reduction is necessary and particularly relevant in elderly patients with osteoporotic bone and comminuted fracture patterns, in whom the goal of osteosynthesis is to re-establish a stable bony framework (‘fix and replace’) [8,10,13]. The anterior inferior iliac spine (AIIS) and the ischium play a vital role in this strategy, as the prosthesis is wedged between these structures; therefore, maintaining a stable relationship between them is essential for implant stability [26,27,28].

Acute THA alone may be appropriate for highly selected fracture patterns in which the structural relationship between the AIIS and the ischium remains intact, most commonly isolated posterior wall fractures or minimally displaced fractures without disruption of the anterior or posterior column continuity. In these scenarios, the remaining subchondral bone can provide adequate support for a press-fit acetabular component, particularly when modern porous, multi-hole cups with supplemental screw fixation are employed [26,27].

In contrast, combined ORIF and THA is indicated when the native acetabular architecture is insufficient to support a stable acetabular component. This includes fractures involving disruption of the anterior column, posterior column, or both, such as anterior column–posterior hemitransverse fractures, both-column fractures, transverse patterns, and T-type fractures. Additional indications for combined fixation include quadrilateral plate medialization, dome or marginal impaction, pelvic discontinuity, and fracture patterns, in which press-fit stability cannot be achieved intra-operatively [15,21,26,29].

Percutaneous or limited internal fixation combined with acute THA has been proposed as a means of restoring columnar stability while minimizing surgical morbidity. Chakravarty et al. demonstrated that selective column fixation using percutaneous screws, followed by immediate THA, provides adequate construct stability and allows early mobilization in elderly patients with acetabular fractures while avoiding the extensile approaches often required for formal ORIF [30]. This strategy is particularly appealing in frail patients, in whom prolonged operative time and blood loss are associated with increased perioperative risk.

Similarly, Beaule and colleagues described the use of the Levine anterior approach to facilitate acute THA in the setting of acetabular fractures, emphasizing that stable implantation of the acetabular component can be achieved without complete fracture reconstruction when the critical osseous buttresses are preserved [27]. Their work highlights that restoration of the relationship between the anterior inferior iliac spine and the ischium is more important for acetabular component stability than precise reduction in all fracture fragments.

Systematic reviews and meta-analyses have reported mean Harris Hip Scores ranging from 80 to 85 following acute THA for acetabular fractures, with revision rates of approximately 4–6% at mid-term follow-up [20,28]. These outcomes compare favorably with delayed THA after failed fixation, which is associated with higher complication and revision rates [14,15]. Contemporary expert consensus also supports the use of acute THA in elderly patients with fracture patterns predictive of fixation failure, particularly when early mobilization is crucial [21,29]. The concept of “traumaplasty” emphasizes definitive joint replacement at the index procedure, analogous to the modern management of displaced femoral neck fractures in frail patients [30]. The concept of cotyloidoplasty, as it has emerged during the recent years, emphasizes the role of arthroplasty not only as a reconstructive device but also as a fixation device, especially when supplemented with femoral autograft and screw fixation, as demonstrated by Chiapale et al. in their case series [31].

The present case series aligns with these principles. By prioritizing implant stability through the use of modern porous acetabular components and selective column fixation—performed in non-anatomic position solely to provide a stable substrate—acute THA enabled early mobilization while avoiding the morbidity associated with staged procedures. The use of femoral autograft and high-porous large cups with screw fixation allowed for reconstruction without the use of allograft or rings, promoting biologic fixation in line with the current literature [31]. As we see in recent adaptations of the Judet–Letournel classification, the integrity of the columns plays the most vital role in our decision-making process in order to assess substrate stability and the need to augment with ORIF or not, following the recent shift in the literature [32].

One should not forget the associated risks involved in the management of these injuries. Even with meticulous planning and evidence-based management, we did not manage to omit two cases of pulmonary embolism, for which high suspicion has to be maintained peri-operatively. An external iliac artery injury was also encountered in a skinny patient, possibly due to a narrower clearance between bone and artery, but the high level of alertness in a tertiary center, including massive transfusion protocols and immediate endovascular stent, ensured the successful outcome for this patient. The above underlines the necessity for meticulous planning, perioperative alertness, a tertiary trauma center, and a surgeon’s expertise in both pelvic fixation and revision hip arthroplasty to ensure a successful outcome in these complex and life-threatening injuries.

### Limitations

This study is limited by its retrospective design, small sample size, and the absence of a comparative control group. Functional outcome scores and long-term implant survivorship were not available for all patients. Nevertheless, the findings are consistent with the growing body of literature supporting acute THA in selected elderly patients with acetabular fractures.

## 5. Conclusions

Acute THA, with or without internal fixation, represents a valuable treatment option for elderly patients with acetabular fractures at high risk of failure following ORIF. When applied judiciously, this strategy enables early mobilization, avoids the need for secondary salvage surgery, and provides acceptable complication and revision rates. Cotyloidoplasty could help fix and replace big acetabular defects at the same time, but it should be used with care as complications are lurking.

## Data Availability

The data presented in this study are available on request from the corresponding author.

## Abbreviations

The following abbreviations are used in this manuscript:

ORIF	Open Reduction Internal Fixation
THA	Total Hip Arthroplasty
mHHS	modified Harris Hip Score
CTPA	Computed Tomography Pulmonary Angiography
AIIS	Anterior inferior iliac spine
